# Assessing the Efficacy of Arthrocentesis in Managing Temporomandibular Joint Disorders and Internal Derangements: A Case Series

**DOI:** 10.7759/cureus.68060

**Published:** 2024-08-28

**Authors:** Munish Kumar, Pritam Pritam, Neeraj Attri, Himanshi Gupta, Mansi Dang

**Affiliations:** 1 Department of Oral and Maxillofacial Surgery, Guru Nanak Dev Dental College & Research Institute, Sunam, IND

**Keywords:** arthrocentesis, case report, corticosteroids, hyaluronic acid, temporomandibular joint disorders

## Abstract

Internal joint derangements (IJD) of the temporomandibular joint (TMJ) encompass a multifaceted array of conditions that have the potential to induce a gradual decline in joint health, ultimately resulting in persistent pain and disruptions in daily living. The management of these conditions poses significant hurdles to achieving effective pain management and restoration of normal TMJ functionality, presenting ongoing challenges in the medical field. At present, the approach of utilizing arthrocentesis in conjunction with intra-articular injections containing corticosteroids (CSs) and hyaluronic acid (HA) is a commonly employed method aimed at addressing chronic pain, surpassing the outcomes of more conservative treatment modalities. Nevertheless, further advancements in treatment strategies are warranted to enhance outcomes and provide holistic relief for individuals with TMJ-related concerns. The effectiveness of arthrocentesis in combination with intra-articular injections of HA and CSs has been demonstrated with the help of five case reports of elderly patients with IJD. All were chronic patients who were unresponsive to conservative management and presented with chronic pain and reduced mouth opening. Open- and closed-mouth magnetic resonance imaging (MRI) provided the diagnosis of IJD, and the patients were treated with arthrocentesis using a two-needle approach that involved flushing the joint space with Ringer’s lactate solution followed by intra-articular injections of 0.5 ml HA and 0.5 ml prednisolone at a concentration of 40 mg/ml into the joint space. All patients showed a significant reduction in pain and improvement in mouth opening at the follow-up visits.

## Introduction

Temporomandibular disorders (TMDs) are clinical problems involving the masticatory musculature, temporomandibular joints (TMJ), and associated structures. It is most common at 20-40 years of age, and females are more affected than males [1]. A recent study suggested that 6%-12% of the global population experiences clinical symptoms of TMDs, with 40%-60% affected in India; however, only 5%-7% have symptoms severe enough to require treatment [2].

The etiology of TMDs is multifactorial (biological, environmental, social, psychological, emotional, and cognitive triggers). Common clinical features of TMDs are pain with mandibular function, earache, headache, facial pain, limitations of mandibular movement, and joint sounds such as clicking and crepitus [3]. Different treatment modalities have emerged and are accepted based on the pros and cons involved, including conservative management, minimally invasive therapy, and surgical intervention [1]. For a long time, meniscectomy was the main surgical treatment for internal joint derangement (IJD) of the TMJ. However, with a better understanding of the intra-articular pathologies and the advent of less destructive treatments such as arthroscopy, the treatment methods have shifted toward minimally invasive approaches [4]. In 1991, inspired by arthroscopy, Nitzan described a technique of lavage of the upper compartment of the TMJ and called it “arthrocentesis,” which has almost similar outcomes to arthroscopy [5]. It is a minimally invasive technique that requires washing the joint to deliver therapeutic agents; it is effective in symptomatic IJD cases and even in the anchored disc phenomenon [6]. The case series aimed to highlight the use of arthrocentesis for the management of TMDs in five case reports.

## Case presentation

Case 1

A 22-year-old woman presented to the Department of Oral and Maxillofacial Surgery (OMFS) with the primary concern of experiencing pain in the vicinity of the right pre-auricular area for four and a half months, accompanied by a decrease in mouth opening. The patient had a history of joint clicking for one year, which ceased following the onset of reduced mouth opening four and a half months ago. The patient reported no history of trauma or parafunctional habits. She did not experience pain while waking up or in other joints. The patient was provided splint therapy for one month, which worsened her condition.

**Figure 1:**
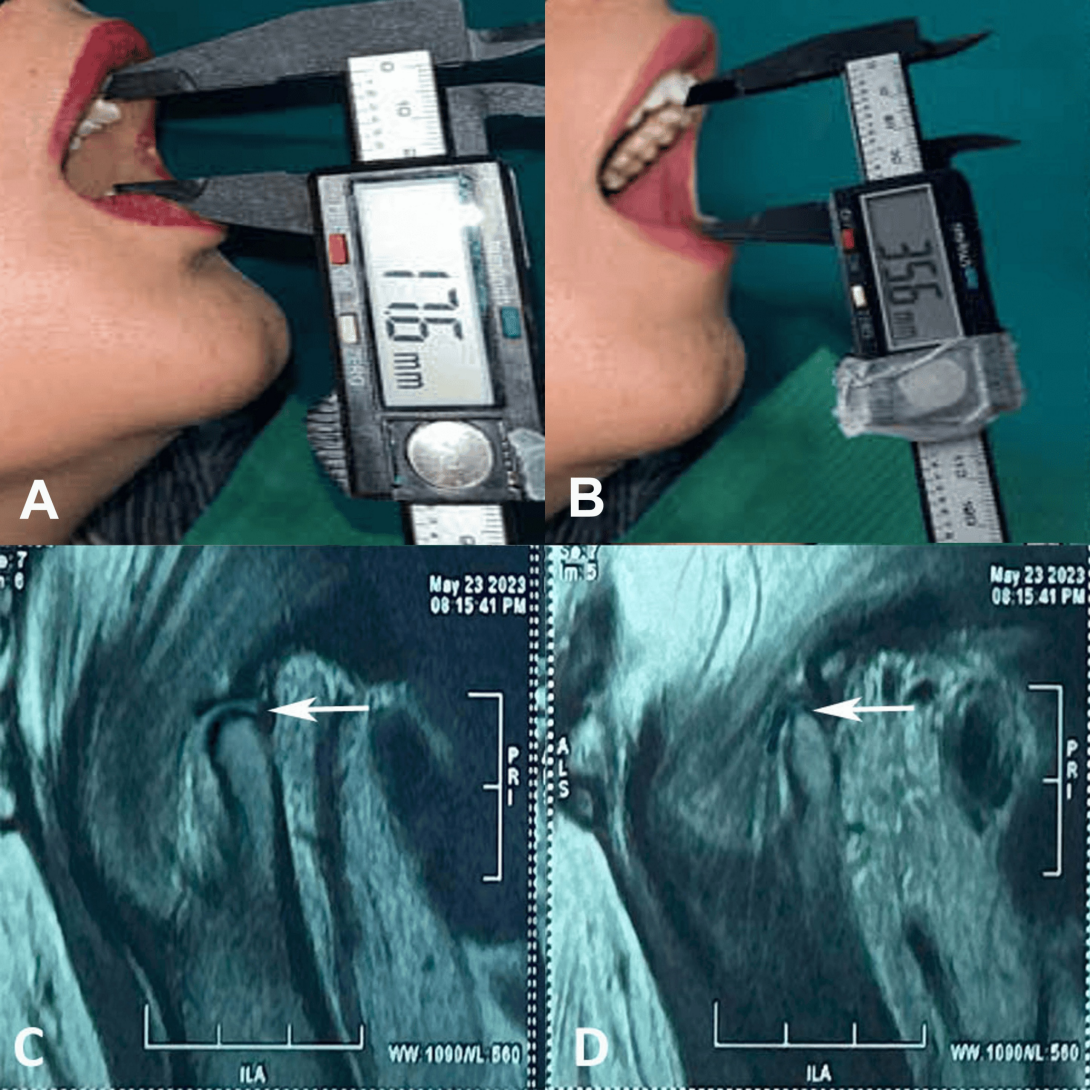
(A) Preoperative mouth opening (17.6 mm), (B) postoperative mouth opening (35.6 mm) at the three-month follow-up, (C) MRI scan with open mouth showing early degenerative changes in the condyle of the right temporomandibular joint, and (D) MRI scan with closed mouth showing anterior disc displacement with reduction of the right temporomandibular joint.

The patient had no relevant medical history. Clinical examination revealed tenderness and deviation of the mandible toward the right side of the mouth opening, restricted lateral excursive movement, reduced mouth opening (17.6 mm), and tenderness on palpation of the right TMJ, right temporalis, lateral pterygoid, and medial pterygoid. No clicking or crepitation was observed. The patient had class 1 malocclusion with lower anterior crowding and a deep bite. Magnetic resonance imaging (MRI) scans were taken in the open and closed positions, and a final diagnosis of anterior disc displacement with reduction of the right TMJ was made. Arthrocentesis was performed using a two-needle technique under local anesthesia (LA) [7]. Through one needle, 100-300 ml of Ringer's lactate was injected into the superior joint space, and the second needle provided an outflow portal that allowed lavage of the joint cavity. It was followed by depositing intra-articular medicaments (0.5 ml hyaluronic acid + 0.5 ml prednisolone 40 mg/ml) into the joint space. The patient was prescribed postoperative analgesics for five days and advised to perform isometric exercises for mouth opening. Mouth opening increased to 26.7 mm at the one-week follow-up and further increased to 35.6 mm at the three-month follow-up (Figure 1).

Case 2

**Figure 2:**
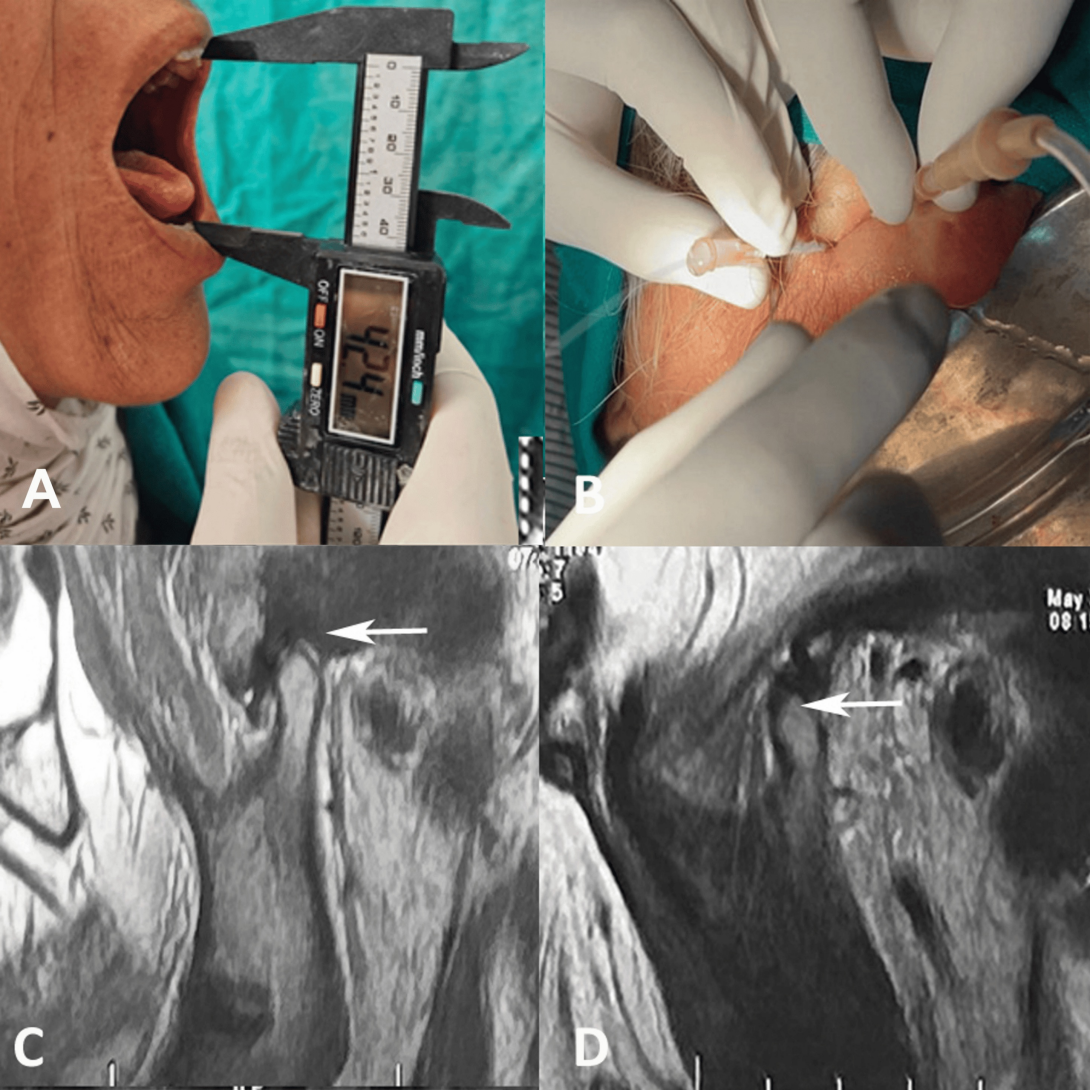
(A) Preoperative mouth opening (42.4 mm), (B) procedure of arthrocentesis of the right temporomandibular joint, (C) MRI scan with open mouth showing synovitis with effusion of the right temporomandibular joint, and (D) MRI scan with closed mouth showing synovitis with effusion of the right temporomandibular joint.

A 66-year-old female reported bilateral pain near the pre-auricular region for one year. The patient provided a record of taking medication to alleviate the pain; however, the symptoms were not fully alleviated. Clinical examination revealed a normal range of motion of the mandible while opening and closing the mouth, and lateral excursion was observed, with a slight deviation of the mandible toward the right when opening the mouth. The mouth opening was 42.4 mm, and tenderness was noted on palpation of the right TMJ. No clicking or crepitation is observed. Open and closed MRI revealed right TMJ effusion with synovitis. The patient was treated with the same protocol as previously described, and on follow-up visits, the patient reported a significant reduction in pain (Figure 2).

Case 3

**Figure 3:**
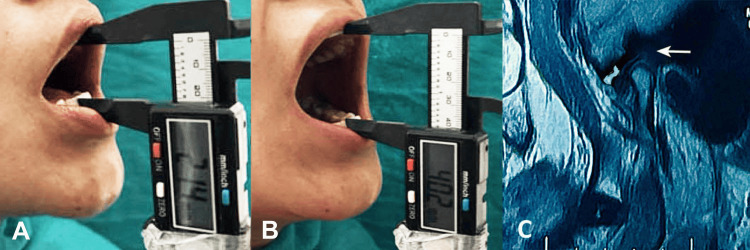
(A) Preoperative mouth opening (21.4 mm), (B) postoperative mouth opening (40.2 mm) at the three-month follow-up, and (C) MRI scan showing right temporomandibular joint effusion.

A 32-year-old female patient reported pain near the right pre-auricular area for eight months. The pain was mild and aggravated, while mouth opening and chewing were associated with reduced mouth opening in the last two months. The patient had a history of multiple visits to various clinicians and had been taking analgesics for pain relief and to improve mouth opening for three years; however, no results were achieved. On clinical examination, reduced mouth opening (14.6 mm) was associated with tenderness on palpation of the right TMJ. No clicking or crepitation was present, and occlusion was normal. Open and closed MRI scans revealed right TMJ effusion, and the patient was treated using the same protocol. At a two-week follow-up, a significant improvement in mouth opening of 21.4 mm was observed, which further improved by 39.7 mm at the three-month follow-up (Figure 3).

Case 4

**Figure 4:**
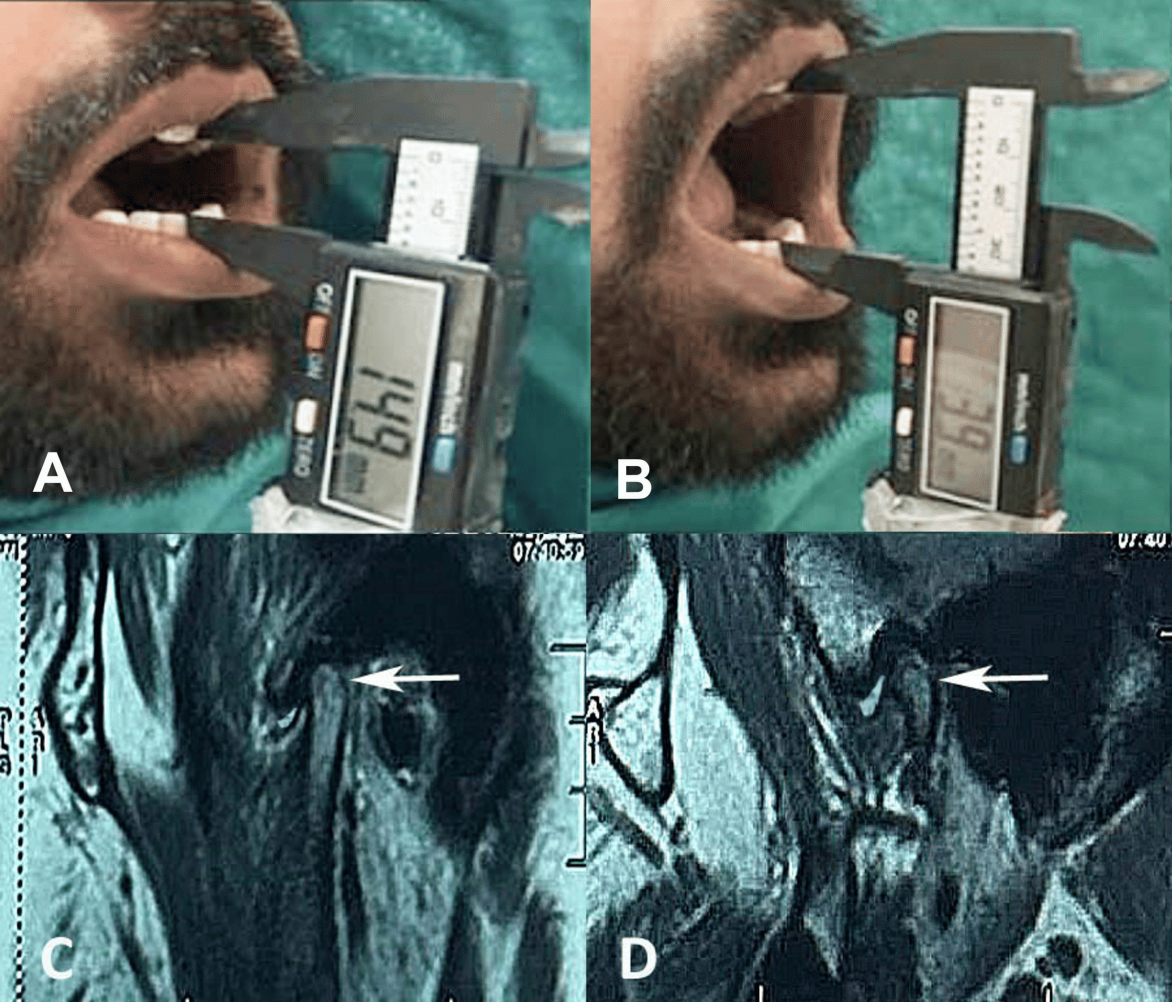
(A) Preoperative mouth opening (14.9 mm), (B) postoperative mouth opening (39 mm) at the three-month follow-up, (C) MRI scan of the closed mouth showing right temporomandibular joint effusion, and (D) MRI scan of the open mouth showing right temporomandibular joint effusion.

A 39-year-old male presented with a chief complaint of pain near the right pre-auricular area for two years. The pain was initially mild and gradually increased over the last four months. The pain was aggravated by chewing, talking, mouth opening, and yawning. The patient had been taking analgesics for the last four months but in vain. No relevant medical history has been reported. On clinical examination, reduced mouth opening (14.9 mm), restricted lateral excursive movement, and tenderness on palpation of the right TMJ were noticed. No clicking or crepitation is observed. Intraoral examination revealed occlusal interference with premature contact between the left upper and lower first premolars. Open and closed MRI scans revealed right TMJ effusion, and the patient was treated with the same protocol after removing occlusal interferences through selective grinding. At the two-week follow-up visit, mouth opening increased to 28.8 mm, and by the three-month follow-up, it further improved to 39 mm (Figure 4).

Case 5

**Figure 5:**
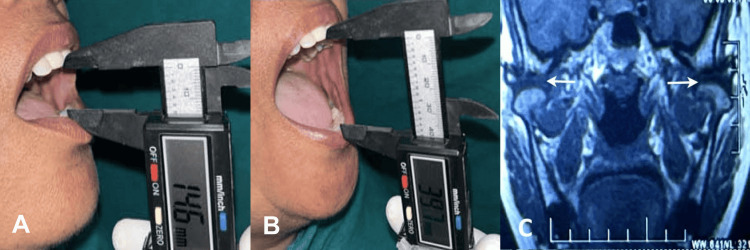
(A) Preoperative mouth opening (14.6 mm), (B) postoperative mouth opening (39.7 mm) at the three-month follow-up, and (C) MRI scan showing partial adhesion of the bilateral temporomandibular joint.

A 46-year-old female patient reported pain bilaterally near the pre-auricular area for six months. The pain was mild and aggravated during chewing and mouth opening. The patient was taking medication for pain relief, which did not completely relieve symptoms. No relevant medical history was reported. Clinical examination revealed a reduced mouth opening of 21.4 mm and tenderness on palpation of the bilateral TMJ, with no clicking or crepitation. The open and closed MRI scans revealed partial adhesion of the bilateral TMJ, which was treated using the same protocol. Mouth opening increased dramatically to 29.2 mm at the two-week follow-up and 40.2 mm at the three-month follow-up (Figure 5).

## Discussion

Almost 10% of the population is affected by TMDs and most commonly affects younger females. IJD is one of the most common TMDs (80% of patients with signs and symptoms of TMDs have some form of IJD) [1]. AAOMS defines IJD as a disruption of the internal aspects of the TMJ. There may be instances where disc displacements or adhesions/impingements occur even when the disc is in a normal position, leading to changes in the typical dynamic movements of the elements within the joint capsule [8]. IJD includes anchored disc phenomenon, disc displacement with reduction, painful click, and closed lock. Patients with IJD of the TMJ often complain of pain, joint sounds, and limited mouth opening [9]. Joint effusion (JE) within the TMJ space is often visualized as heightened signal intensity on T2-weighted MRI. JE has been proposed as a potential indicator of intra-articular inflammation in individuals with IJD of the TMJ. Moreover, JE is widely accepted as a hallmark sign of synovitis in cases of IJD, suggesting an inflammatory process within the joint space [10].

JE has been linked to TMJ pain, which was also observed in our patients [11]. TMJ pain has been linked to joint fluid because changes in the quantity of joint fluid have been correlated with TMJ pain [11]. About 90% of TMDs demonstrate resolution either spontaneously or through non-surgical treatment, which can be attributed to the underlying adaptive capacity of the TMJ. Notably, a subset of patients, ranging from 25% to 33%, who do not exhibit improvement even after a year, are typically older individuals, particularly those with MRI findings indicating advanced disease progression. Surgical intervention is a viable option in such cases. However, before considering surgery, non-surgical therapies are recommended to be pursued for one to two months in most cases to explore all possible avenues of treatment and to ensure the best possible outcome [2,3]. Patients who do not exhibit a positive response to conservative treatment methods may find themselves in need of more aggressive interventions, including arthrocentesis and arthroscopy [5]. Initially, the process of irrigating the TMJ was performed through arthroscopic means; however, it was later realized that direct visualization of the joint may not be essential for achieving therapeutic goals. Consequently, the focus shifted toward utilizing arthrocentesis as a standalone technique, serving as a modification of the previous arthroscopic lavage approach.

The effectiveness of arthrocentesis in alleviating pain aligns with the concept that joint lavage aids in the elimination of proinflammatory cytokines and degradation by-products responsible for synovitis, thereby supporting the reduction of pain experienced by the individual [12]. To enhance the extent of mouth opening and alleviate discomfort in our patients, arthrocentesis was performed by inserting two catheters into the upper joint area while the patient was under the influence of LA, coupled with irrigation using Ringer’s lactate solution. This therapeutic approach has demonstrated significant efficacy in addressing disc displacement without reduction, particularly in cases involving restricted mouth opening, such as those characterized by a closed-lock condition. Compared to alternative surgical interventions targeting the TMJ, arthrocentesis is effective in reducing pain and promoting increased mouth opening, with a notably low occurrence rate of associated complications [13]. Similar results of increased mouth opening and alleviation were also reported in a systematic review by Siewert-Gutowska et al. [14].

Hyaluronic acid (HA) plays a significant role in determining the viscosity of a typical synovial joint, contributing to its lubricating properties. Furthermore, its ability to act as a molecular sieve is believed to have crucial implications in the regulation of the nutritional environment around the articular cartilage and the physical interactions with the macromolecules located on the articular surfaces [15]. Corticosteroids (CSs) induce a wide range of effects on both the cellular and humoral immune systems, eliciting significant anti-inflammatory and immunosuppressive reactions. These effects are profound and influence various aspects of the immune response within the body. Furthermore, when CSs are locally injected, they exhibit a strong anti-inflammatory effect, specifically targeting the synovial tissue, leading to the reduction of effusion and alleviation of pain, ultimately resulting in an enhanced range of motion in the affected joint [16]. A combined lavage of the TMJ by CSs and HA during arthrocentesis leads to significant improvement in mouth opening and alleviation of pain, as observed in our study, which was in accordance with previous studies [17,18]. A study by Dhiman et al. reported significant improvement in mouth opening and pain with the combined approach of HA and CSs in the management of TMD, with the results maintained at six months of follow-up [17]. Similar results were reported by Bjørnland et al., where HA or CSs led to significant improvement in pain where patients were suffering from TMJ pain [18].

The strength of our case series lies in the successful management of the patient with arthrocentesis with lavage of HA and CSs, which led to significant improvement in the chronic non-refractory TMJ pain. However, the major limitation of our case series was the short-term follow-up of three months.

## Conclusions

This article has illustrated through a series of cases that arthrocentesis combined with the infusion of HA and CSs represents a valid and relatively more efficient approach for managing IJD in patients who are chronic and unresponsive to conservative therapies. A significant reduction in discomfort and enhancement in jaw functionality following the treatment were observed after the procedure. Despite being a somewhat invasive delivery method, infiltration has proven to be highly dependable when performed accurately. This minimally invasive technique has the potential to become increasingly prominent in the field of OMFS, serving as a viable alternative for addressing chronic TMJ pain in individuals who do not respond well to conservative medical intervention.
